# Modeling and Optimization of Phenolic Compounds from Sage (*Salvia fruticosa* L.) Post-Distillation Residues: Ultrasound- versus Microwave-Assisted Extraction

**DOI:** 10.3390/antiox12030549

**Published:** 2023-02-21

**Authors:** Maria Irakli, Elisavet Bouloumpasi, Stamatia Christaki, Adriana Skendi, Paschalina Chatzopoulou

**Affiliations:** 1 Hellenic Agricultural Organization—DIMITRA, Institute of Plant Breeding and Genetic Resources, Thermi, 57001 Thessaloniki, Greece; 2 Department of Food Science and Technology, School of Agriculture, Aristotle University of Thessaloniki, 54124 Thessaloniki, Greece; 3 Department of Food Science and Technology, International Hellenic University, 57400 Thessaloniki, Greece

**Keywords:** optimization, microwave-assisted extraction, ultrasound-assisted extraction, phenolic antioxidants, *Salvia fruticosa*, post-distillation, antioxidant activity, rosmarinic acid, carnosol, carnosic acid

## Abstract

The essential oil production of *Salvia fruticosa* L. generates considerable amounts of post-distillation solid residues (PRES) which are rich in phenolic compounds. In the present work, the recovery of phenolic antioxidants from PRES by using Microwave-Assisted Extraction (MAE) and Ultrasound-Assisted Extraction (UAE) were separately optimized, according to the Box–Behnken experimental design. The optimization was based on extraction yield, total phenolic content (TPC), total flavonoid content (TFC), rosmarinic acid (RMA), carnosol (CARO), carnosic acid (CARA), and antioxidant activity. The optimal processing parameters were 72% and 68% ethanol, a 15- and 10-min extraction time, a 40 °C and 47 °C extraction temperature, and a 1:30 and 1:10 solid-to-solvent ratio, for MAE and UAE, respectively. Results showed that the levels of RMA, CARO, and CARA in UAE extracts were influenced mainly by ethanol concentration, extraction time, and extraction temperature, while MAE extracts were only influenced by the first two factors. Experimenting with the optimal conditions revealed MAE as more effective than UAE in the recovery of RMA and CARA. The experimental values were in good agreement with the predicted ones, indicating model efficacy in MAE and UAE optimization to effectively extract phenolic compounds from PRES for their further application in food and pharmaceutical industries.

## 1. Introduction

The current demand of the global market for the sustainable production of food products is pushing the food industry and researchers towards a major change regarding both raw materials and eco-friendly production practices. In this respect, the interest in the exploitation of aromatic and medicinal plants (MAPs) as sources of natural bioactive compounds is steadily rising because of the increased uses of their derivatives (essential oils and extracts) as antioxidants and antimicrobial agents in food products. Apart from the herbal plant material, a new trend, in terms of the circular economy, is the valorization of MAPs by-products from the essential oil industry (post-distillation residues) for the recovery of phenolic compounds with important biological activity. In addition, the development and implementation of sustainable extraction techniques, resulting in green extraction methods, are widely accepted because of their high efficiency and lower cost compared to conventional methods, i.e., Soxhlet and maceration [1,2]. The novelty of the newly developed methods is the minimization of environmental impact by lowering the extraction time and solvent and energy consumption as well as maximization of the yield of the targeted bioactive compounds, i.e., polyphenols [3,4]. 

Green extraction technologies such as Microwave-Assisted Extraction (MAE) and Ultrasound-Assisted Extraction (UAE) have been considered as potential alternatives to the conventional extraction of bioactive components from plant materials. MAE utilizes the energy of microwaves to disrupt the hydrogen bonds of polar molecules in the extraction system by rapidly rotating them through ion conduction or dipole-dipole rotation [5], resulting in a high extraction rate, short extraction times, superior product quality, and low solvent requirements [6]. This is why this method is highly recommended for extracting short-chain polyphenols, such as flavonoids and phenolic acids, from plant materials. Novel developed commercial MAE equipment provides the opportunity for the simultaneous extraction of multiple samples, thus limiting energy consumption to a greater extent. MAE has been used as an efficient method for the recovery of polyphenols from a variety of plant materials, including MAPs [3,7,8]. Among the main parameters affecting the polyphenols’ extraction employing MAE are the extraction time and temperature, the irradiation power, and the dielectric constant of the solvent (polarity) [9]. Consequently, these factors affect the efficiency in terms of the recovery of the bioactive compounds. 

On the other hand, UAE utilizes the mechanism/principle of acoustic cavitation, where mechanical energy produced through circles of compression and rarefaction is transferred via ultrasonic waves to produce nanobubbles. When the nanobubbles’ energy exceeds their resistance threshold, they collapse, leading to the destruction of plant cell walls, facilitating solvent diffusion within the plant cells that, in turn, results in an increase in bioactive compound mass transfers [10]. UAE outperforms conventional methods due to its mild extraction conditions (time and temperature), simplicity of use, economic benefits, and high extraction efficiency. Similar to MAE, the main parameters affecting the efficiency of UAE are the extraction temperature and time, solvent characteristics, and ultrasound power and frequency [11]. Various studies have compared the effectiveness of these two extraction methods in different plant materials [12,13,14,15,16] or by-products [5,6,10], including a by-product of *S. officinalis* (sage herbal dust) [3]. Unlike UAE, the recovery of compounds from the post-distillation residues of MAPs employing MAE is not as thoroughly investigated in the literature.

Among MAPs, sage is one of the largest and most valued genera of the Lamiaceae family, comprising over 800 species worldwide. Several species are known for their medicinal properties and have been used in traditional medicine since antiquity. Sage is a natural source of polyphenols, which possess antioxidant and radical scavenging properties [17]. Recently, phenolic compounds have received considerable attention due to their protective role against oxidative stress and free radical-induced damage, which are associated with chronic diseases such as diabetes, cardiovascular disease, cancer, heart disease, brain dysfunction, etc. [18]. Lopresti [19] summarized the current knowledge and potential effect of plants belonging to the genus *Salvia* on cognitive skills, including memory, attention, and neurodegenerative diseases. In addition, modern studies have shown numerous pharmacological properties owed to the presence of several bioactive compounds, such as essential oils and polyphenols.

*Salvia fruticosa* Mill., commonly known as Greek sage, is an important plant of the Mediterranean area and is mainly found in coastal areas throughout Greece [20,21]. Methanolic extracts of *Salvia fruticosa* contain phenolic acids (e.g., caffeic acid and rosmarinic acid), flavonoid glycosides (luteolin, nepetin, apigenin, luteolin-*O*-glucuronide, luteolin-*O*-glucoside, and apigenin-*O*-glucuronide), rosmanol isomers, carnosic acid, carnosol, ursolic acid, and stearic acid, among others [2,4,22,23]. A few green extraction processes employing ultrasonication pre-treatment combined with deep eutectic solvents [1,21] and cyclodextrin solutions [24] have been developed for the production of polyphenol-rich extracts from *S. fruticosa*. Although essential oil production generates considerable amounts of post-distillation solid residues, there are no studies on the recovery of phenolic compounds from these by-products using MAE or UAE methods. Both technologies are recommended as ‘green’ and efficient for the valorization of essential oil by-products. However, there is inadequate data on the optimization of extraction conditions and the effect of MAE and UAE parameters on the phenolic antioxidant compounds of post-distillation solid residues.

Thus, the present study aimed to determine the optimal extraction conditions for the recovery of antioxidant phenolic compounds from the post-distillation solid residue of *Salvia fruticosa* (PRES) utilizing two green extraction techniques, UAE and MAE. For this purpose, a comparative optimization study was performed on the extraction parameters of both methods regarding the extraction efficiency of phenolic compounds and the antioxidant activity of the extracts. Response Surface Methodology (RSM) was used to optimize the extraction conditions as the established models evaluated and compared the effects of the dependent variables using quantitative results. 

## 2. Materials and Methods

### 2.1. Plant Material 

Aerial parts of *Salvia fruticosa* L. were collected from cultivated accessions of the Hellenic Agricultural Organization—DIMITRA, Institute of Plant Breeding and Genetic Resources (Thermi, Thessaloniki, Greece) and were sun-dried. The dried material was subjected to steam distillation in a pilot-scale essential oil distillation apparatus for approximately two hours. The wet solid residue of *Salvia fruticosa*, a by-product of post-distillation (PRES), was collected and then sun-dried for 48 h. The dried material (~10% moisture content) was then ground in a laboratory mill (Retsch, Model ZM 1000, Haan, Germany) to pass through a 0.5 mm sieve and stored at 4 °C until further analysis.

### 2.2. Chemicals and Reagents

The analytical reagents 2,2-azinobis-(3-ethylbenzthiazoline-6-sulphonic acid) (ABTS), 2,4,6-tripyridyl-s-triazine (TPTZ), and 2,2-diphenyl-1-picryhydrazyl (DPPH) radical were procured from Sigma-Aldrich (Steinheim, Germany). Analytical standards of rosmarinic aid (RMA), gallic acid (GA), and catechin (CAT) were purchased from Extrasynthese (Genay, Cedex, France), whereas carnosol (CARO) and carnosic acid (CARA) were obtained from Carbosynth (Compton, Berkshire, United Kingdom). All the solvents used for extraction and chromatographic analysis were HPLC (High Performance Liquid Chromatography) or LC-MS (Liquid Chromatography – Mass Spectrometry) grade.

### 2.3. Green Extraction Methods

#### 2.3.1. Ultrasound-Assisted Extraction (UAE)

All UAE experimental runs were carried out using an ultrasonic probe (Sonoplus model HD 4100, Berlin, Germany) consisting of an ultrasonic generator GM 4200, an ultrasonic converter UW 100, and a titanium probe TS 103, diameter 3 mm. The frequency was set at 20 kHz under a working amplitude equal to 50%. The device was used throughout the experiment with a pulse length of 2 s and an interval of 0.5 s. Extracts were prepared by mixing dried and milled PRES samples (0.67, 1.00, and 2.00 g) with 20 mL ethanol (0, 40, and 80%) in a 50 mL plastic centrifuge tube, and extracted by an ultrasonic probe which was immersed 1 cm deep into the mixture at varying levels of extraction time (2, 6, and 10 min) and extraction temperatures (30, 45, and 60 °C). The temperature of the mixture inside the extractor was monitored using a thermocouple attached to the sonicator system. After extraction, the extracts were filtered through Whatman filter paper no. 1, and the filtrates were centrifuged (Universal 320R, Hettich, Frankenberg, Germany) at 4000 rpm (2680× *g*) for 10 min. The supernatants were evaporated by using a rotary evaporator (Heidolph Instruments GmbH & Co. KG, Schwabach, Germany) at 40 °C under a vacuum in order to remove the ethanol from the extract. The remaining aqueous extract and the washings were subjected to freeze drying in a laboratory freeze-dry system (Christ, Martin Christ Gefriertrocknungsanlagen GmbH, Germany) for 48 h. The dried extracts were weighed and stored at •25 °C until subsequent analyses. The extraction yield (EY) of the phenolic extracts of PRES was calculated using the gravimetric method according to the following equation:

(1) $$\mathrm{EY}\;\left(\%\;w/w\right)=\frac{\mathrm{weight}\;\mathrm{of}\;\mathrm{extract}\;\mathrm{recovered}\;\mathrm{after}\;\mathrm{lyoplilization}\;\left(g\right)\;}{\mathrm{weight}\;\mathrm{of}\;\mathrm{dry}\;\mathrm{raw}\;\mathrm{material}\;\mathrm{of}\;\mathrm{PRES}\;\left(g\right)}\times 100$$

Then, the freeze-dried extracts were stored at −20 °C to be used for the spectrophotometric and LC-MS analyses. For analysis, 0.0050 g of each dried extract was re-dissolved in 4 mL 70% methanol and the solutions were filtrated through PTFE syringe filters (13 mm diameter, pore size 0.45 μm pore size).

#### 2.3.2. Microwave-Assisted Extraction (MAE)

The MAE experiments were performed using a commercial ETHOS X microwave-assisted extraction system (Milestone, ETHOS X, Sorisole, Italy). The system was equipped with two industrial magnetrons (950 W each) that performed the microwave irradiation, and a high-efficiency extraction rotor inside the sample chamber with 15 positions (extraction units). All the parameters, such as temperature, time, and power, were controlled by the respective software panel (terminal). The extractions were performed using lid-covered TFM vessels (100 mL max. volume), with safety shields, that were individually placed in rotor bodies. These segments were subsequently placed into the rotor (turntable plate). For the extractions, dried and powdered PRES were mixed with 20 mL aqueous ethanol solutions (0, 40, or 80%) at different solvent-to-solid ratios (10, 20, and 30 mL/g). The experiments were conducted at atmospheric pressure and 600 W under a heating-cooling cycle composed of different extraction times (3, 9, or 15 min) and temperatures (40, 65, and 90 °C). The temperature inside the vessels was monitored via an infrared easyTEMP sensor that was placed on the bottom of the microwave cavity. The cooling cycle for each treatment that followed extraction lasted 10 min, with the samples being cooled by the cooling fan attached to the unit. After completion of the extraction process, the extracts were treated as described in Section 2.3.1.

### 2.4. Analytical Determinations of Phenolics and Antioxidant Activity 

#### 2.4.1. Total Phenolic Content (TPC)

For the determination of the TPC of the samples, the Folin–Ciocalteu assay was used according to the protocol described by Singleton et al. [25]. Briefly, 0.2 mL of the UAE and MAE extracts and 0.8 mL of the Folin–Ciocalteu reagent (diluted 10-fold in deionized water) were mixed with 2 mL of sodium carbonate (7.5 % w/w). Deionized water was added until the final volume was 10 mL, and then the samples were incubated for 60 min at room temperature and the absorbance was measured at 725 nm. Tests were carried out in triplicate, and the results were expressed as mg gallic acid equivalent (GAE) per g of extract (mg GAE/g).

#### 2.4.2. Total Flavonoid Content (TFC)

The TFC of the samples was determined according to the aluminum chloride colorimetric method by Bao et al. [26], with slight modifications. Briefly, 0.3 mL of UAE or MAE extract was mixed with 1.5 mL of deionized water and 0.225 mL of 5% sodium nitrite solution. After 5 min, 0.225 mL of 10% aluminum chloride hexahydrate was added. The mixture was allowed to rest for 5 min before adding 0.75 mL of 2 M sodium hydroxide. Finally, after 20 min, the absorbance was measured at 510 nm using a spectrophotometer. The tests were performed in triplicates, and the results were expressed as mg of catechin equivalents (CATE) per g of extract (mg CATE/g).

#### 2.4.3. DPPH Radical Scavenging Activity 

The antioxidant activity of the extracts was evaluated using the DPPH assay as described by Skendi et al. [27]. Briefly, 150 μL of the diluted extract was mixed with 2.85 mL of fresh prepared DPPH^•^ methanolic solution (0.1 mM). The mixture was incubated for 5 min at room temperature under darkness and the absorbance was measured at 516 nm. The DPPH solution was used as a control. Inhibition of DPPH^•^ (%) was calculated by using the following equation:

(2) $$Inhibition\;\left(\%\right)=\left[\frac{A0-As}{A0}\right]\times 100$$

where *A*0 is the absorbance of the blank sample and *As* is the absorbance of the extract at 5 min. The experiment was carried out in triplicate and the DPPH radical scavenging activity of the extract was calculated and reported as mg Trolox equivalent per g of extract (mg TE/g).

#### 2.4.4. ABTS Radical Scavenging Activity 

The radical scavenging activity of the extracts against the ABTS radical cation was evaluated according to the protocol of Re et al. [28] and appropriately adjusted. Briefly, ABTS + solution was obtained by mixing 2 mmol/L ABTS stock solution with 0.73 mmol/L potassium persulfate and the mixture was left to stand in the dark at room temperature for 12–16 h before use. The ABTS + solution was diluted with water to an absorbance of 0.70 ± 0.02 at 734 nm. After the addition of 100 µL of phenolic extract to 3.9 mL of diluted ABTS + solution, the absorbance was measured against a blank at 734 nm after 4 min. Inhibition of the ABTS radical cation (%) was calculated as described in Section 2.4.3. 

### 2.5. Quantification of Phenolic Compounds by LC-DAD-ESI-MS 

Quantification of the main phenolic compounds presented in PRES, such as RMA, CARO, and CARA, was performed on a Shimadzu Nexera HPLC system (Shimadzu, Kyoto, Japan) equipped with a diode array detector (DAD), and a single quadrupole mass spectrometer combined with an electrospray ionization (ESI) interface, according to the chromatographic conditions as described by Irakli et al. [29]. The separation of phenolic compounds was achieved on a Poroshell 120 EC-C_18_ column (4.6 × 150 mm, 4 μm) thermostated at 35 °C. The composition of the mobile phase was aqueous formic acid (0.1%, *v*/*v*) (solvent A) and acetonitrile (solvent B), with a flow rate of 0.5 mL/min. Gradient elution was performed as follows: 0–5 min, 15–25% B; 5–10 min, 25–35% B; 10–28 min, 35–60% B; 28–35 min, 60–100% B; and 35.01–40 min, 100–15% B and an isocratic elution until 45 min. The injection volume was 10 μL and the quantitative determination of target compounds in the phenolic extracts was carried out in negative ion and SIM mode, constructing calibrations curves of corresponding standard solutions at five concentration levels within the linear range of 0.01 to 10 μg/mL for RMA and 0.05 to 200 μg/mL for CARO and CARA. Data acquisition and processing were performed using Lab Solutions LC-MS software version 5.97.SP1 (Shimadzu, Kyoto, Japan). Analyses were performed in triplicate and the results were expressed as mg per g of extract. 

### 2.6. Design of Experiments and Statistical Analysis

The influence of the process parameters on MAE and UAE was investigated using RSM, adopting a three-level, four-factor Box–Behnken experimental Design (BBD). Minitab statistical software version 18 (Minitab Inc., State College, PA, USA) was used to establish a mathematical model and obtain the optimum conditions for the maximum recovery of phenolic compounds from PRES. Independent variables including ethanol concentration (X_1_), extraction time (X_2_), extraction temperature (X_3_), and solvent-to-sample ratio (X_4_) were then selected. According to the BBD and keeping the independent variables at three levels (−1, 0, and +1), a total of 27 experimental runs were conducted to determine the EY, TPC, TFC, RMA, CARO, CARA, and antioxidant activity (ABTS and DPPH), including three replicates with a random combination of independent variables. The coded and uncoded values of factors at the three levels are given in Table 1. The levels of independent variables for the extraction of the polyphenols were selected based on the results obtained from our preliminary experiments. For the dependent variables, a full quadratic mathematical model was created using the multiple regression analysis method. The significant terms in the model were found using variance analysis (ANOVA), taking into account a probability value of 0.05. The non-significant coefficients of the model were omitted from the equation. The accuracy of the model was evaluated by lack-of-fit and the Fisher test value (F-value). The fit of the model was evaluated by the coefficient of determination (R^2^), the adjusted determination coefficient R^2^ (adj), and the predicted coefficient R^2^ (pred).

For each response variable, a second-order polynomial equation was determined separately for MAE and UAE as:

(3) $$Y=\beta 0+\overset{k}{\underset{i=1}{\sum }}\beta iXi+\overset{k}{\underset{i=1}{\sum }}\beta iiXi^{2\;}+\overset{k}{\underset{i=1}{\sum }}\overset{k-1}{\underset{j=i+1}{\sum }}\beta ijXiXj$$

where *Y* is the response variable, *β*0 is the intercept, *βi* is the linear regression coefficient for the *i^th^* factor, *βii* for quadric, and *βij* for the cross-product term. *Xi* and *Xj* are the independent variables and *k* is the number of tested variables (*k* = 4). 

Three-dimensional response surface plots were generated for each response by keeping two independent variables constant at medium levels and plotting the other two independent factors with the response, in order to visualize the relationship between the response and experimental levels of each factor (Design Expert trial version 11.0 Stat-Ease Inc., Minneapolis, MN, USA). 

BBD experimental data was used to find the optimized conditions for the independent variables (X_1_, X_2_, X_3_, and X_4_). The optimization by Minitab software was performed through the desirability function. The desirability for response variables (EY, TPC, TFC, RMA, CAR, CARA, and antioxidant activity) was kept at maximum and the independent variables were explored within their range of values (between the lowest and the highest level), giving the highest combined desirability (D). Differences in the mean values of the responses between MAE and UAE were tested with the Tukey test (*p* ≤ 0.05) using Minitab software version 18 (Minitab Inc., State College, PA, USA).

## 3. Results and Discussion

### 3.1. Modeling, Fitting, and Adequacy of the Models for MAE and UAE

The selection of an optimized extraction method to recover phenolic compounds from plant materials has gained particular interest in recent years due to the current trend to valorize agri-food by-products [30]. Consequently, efforts have been made to develop efficient, sustainable, “green” extraction methods that increase both yield and antioxidant capacity [31]. In this study, the suitability of MAE and UAE to recover phenolic compounds from PRES was investigated and optimized by RSM. RSM is a valuable optimization tool used for developing, improving, and optimizing processes in which responses of interest are influenced by several variables [32]. 

The variables of solvent concentration (X_1_), time (X_2_), temperature (X_3_) of extraction, and solvent-to-solid ratio (X_4_) were chosen in order to optimize the extraction process for MAE and UAE. According to Zhang et al. [33], the extraction of secondary metabolites from plants and agro-industrial by-products using microwaves can be affected by the solvent, solvent-to-raw material ratio, power and irradiation time, particle size, temperature, and the number of extraction cycles. Similarly, relevant parameters that could enhance the solubility of phenolic compounds with the increase in mass transfer and reduction in solvent viscosity through ultrasounds are temperature, time, solvent, frequency, and ultrasonic power [34]. The selection of the extraction solvent is important because it plays a critical role in the amount and type of phenolic compounds extracted, depending on the plant matrices or extraction techniques applied [35]. Ethanol–water mixtures were used in the present study for the recovery of phenolic extracts from PRES because they are classified as sustainable and safe solvents [36]. 

**Figure 1:**
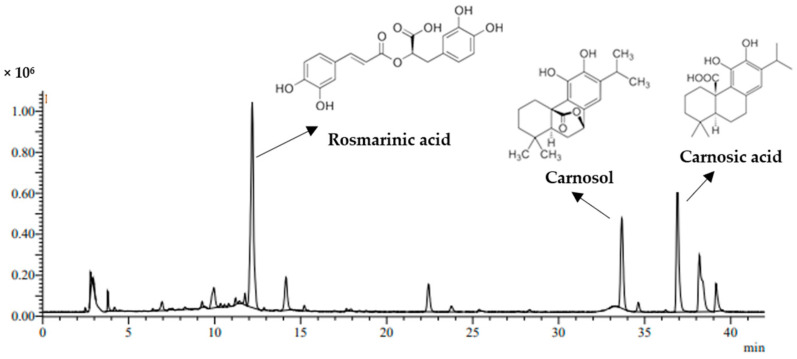
TIC profile of sage solid residue extract with the chemical structures of rosmarinic acid, carnosic acid, and carnosol, recorded in the negative ion mode for UAE under optimized conditions.

The selection of the variable ranges applied for the MAE and UAE of the phenolic antioxidants was based on preliminary experiments. The response design for polyphenol extraction from PRES using both MAE and UAE comprised a total of 27 runs, generated according to the Box–Behnken model (Table 2). The same ranges of ethanol concentration (0–80%) and solvent-to-solid ratio (10–30 mL/g) were used for both MAE and UAE, however, different times and temperatures were selected according to preliminary tests; 3–15 min or 2–10 min and 40–90 °C or 30–60 °C, for MAE and UAE, respectively. EY, TPC, TFC, antioxidant activity, and the main phenolic compounds concentrations as determined by LC/MS were used as responses in the RSM experimental design. The chemical profile of PRES polyphenols has been investigated in detail and it has been reported that RMA (phenolic acid), CARO, and CARA (phenolic diterpenoids) were the most abundant compounds [4,29]. A representative chromatograph of the phenolic compounds extracted under the optimized UAE conditions is illustrated in Figure 1. The identification of the main peaks was confirmed by comparing their retention time values with those of the reference compounds, according to the data in Table 3. 

The effect of independent variables on the MAE and UAE process-dependent variables was investigated based on the statistical analysis for each response (Table S1). The regression equations that explain each response were generated to contain only statistically significant terms (*p* ≤ 0.05). The quadratic polynomial model equations for each response in uncoded units together with statistical analysis are presented in Table 4. The regression coefficients of the developed models demonstrated significant (*p* ≤ 0.05) relationships between the extraction process (MAE and UAE) variables and the corresponding responses of the obtained extracts. All the fitted models showed a coefficient of determination (R^2^), adjusted determination coefficient (R^2^_adj_), and predicted coefficient (R^2^_pred_) higher than 0.61, 0.58, and 0.50, respectively. The lowest fit was observed for the TFC response obtained from the MAE process. Some differences between the two chosen extraction processes were observed. The R^2^ as well as the R^2^_adj_ and R^2^_pred_ of the developed models for MAE were lower than the respective values for UAE. However, comparing the R^2^_pred_ with the R^2^_pred_ for each equation generated (less than 0.13) revealed a very good prediction ability. In addition, the equations obtained for each response have a lack-of-fit value ranging from 1.35 to 11.64 and *p* > 0.05. The insignificance of the lack-of-fit test (*p* > 0.05) verified the suitability of the generated model for all responses. 

### 3.2. Effect of Extraction Factors on Experimental Responses Using MAE

According to the results of Table 4, all the MAE responses were affected by the linear term of ethanol concentration, as it is confirmed by the significant coefficients of this independent factor (*p* ≤ 0.05). 

All responses except for EY were affected positively by the ethanol concentration, suggesting that higher levels of ethanol favor the recovery of the phenolics and produce extracts with high antioxidant activity but low yield. The literature data underline the high influence that the ethanol percentage has on the extraction of phenolics from plant materials, such as *Rosmarinus officinalis* [37] or sage by-products [3]. A significant negative quadratic effect was observed only for TPC, ABTS, DPPH, and CARO. This suggests that the positive effect in increasing TPC and CARO levels, and antioxidant activity of the extracts (ABTS and DPPH), decreases at higher ethanol concentrations, whereas in the case of CARO and DPPH, this positive effect is reversed at ethanol concentrations higher than 55% (Supplementary Figure S1). These findings suggest that the use of high ethanol levels is not efficient in recovering CARO with high antioxidant activity. Only one interaction effect was observed for ethanol: a positive interaction with time was observed for RMA response.

A positive and moderately significant influence of extraction time was noticed only in the ABTS radical scavenging activity of the PRES extracts (*p* ≤ 0.05), whereas a significant negative influence in EY was observed. Extraction time had no significant influence on other responses. As previously noted, the interaction between ethanol and time has an impact on the RMA response. To improve the recovery of RMA, it is suggested that the lowest extraction time be used at an ethanol concentration of less than ~35% and the highest extraction time at higher concentrations (Supplementary Figure S1). The negative quadratic effect of time, which only influenced EY, showed that EY declined with increasing extraction time up to about 8 min, but then this pattern is reversed. 

The negative influence of the linear term of temperature in the above phenolic compounds (RMA, CARO, and CARA) was the most noticeable effect, which is rather expected since phenolic compounds undergo thermal degradation with increased microwave temperature [38]. However, Jacotet-Navaro et al. [39] found that RMA does not appear to be a thermo-sensitive compound, compared to CARA, when MAE was applied. In addition, temperature exercises a significant positive quadratic effect in the case of EY and a negative in the case of DPPH. Therefore, EY decreases with increasing temperature until about 60 °C and after that increases, whereas the opposite was observed for DPPH. 

Optimizing the EY is critical to the development of antioxidant extracts, as increasing EY can reduce the overall production cost. The EY obtained by applying MAE was highly positively influenced by the solvent-to-solid ratio linear effect. Combining this effect with that of the solvent concentration, time, and temperature of extraction suggested that extending the MAE time above 10 min, a MAE temperature above 70 °C, and a solvent-to-solid ratio above 20 could result in a continuous increase in EY. In addition, TPC was affected by the positive linear effect of the solvent-to-solid ratio, whereas the other responses were not affected by this factor. Only CARA was influenced by the quadratic effect of the solvent-to-solid ratio. CARA levels were decreased when the solvent-to-solid ratio was increased to 20 but for higher values, the trend was reversed. Various extraction studies have reported that increasing the solvent-to-solid ratio can facilitate the mass transfer of compounds from the plant matrix into the solvent [40,41]. 

**Figure 2:**
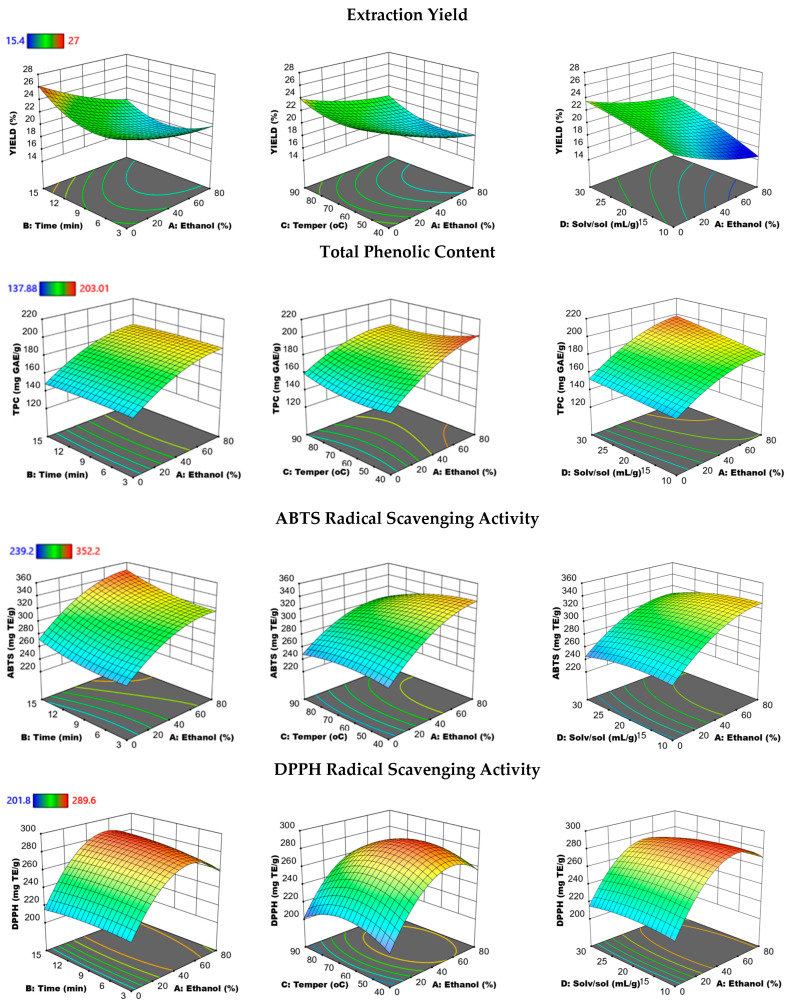
Response surface plots showing the combined effects of the MAE parameters (% ethanol-time, % ethanol-temperature, and % ethanol solvent-to-solid ratio) on the extraction yield, total phenolic content, ABTS, and DPPH radical scavenging activity by keeping the two independent variables constant at medium levels.

**Figure 3:**
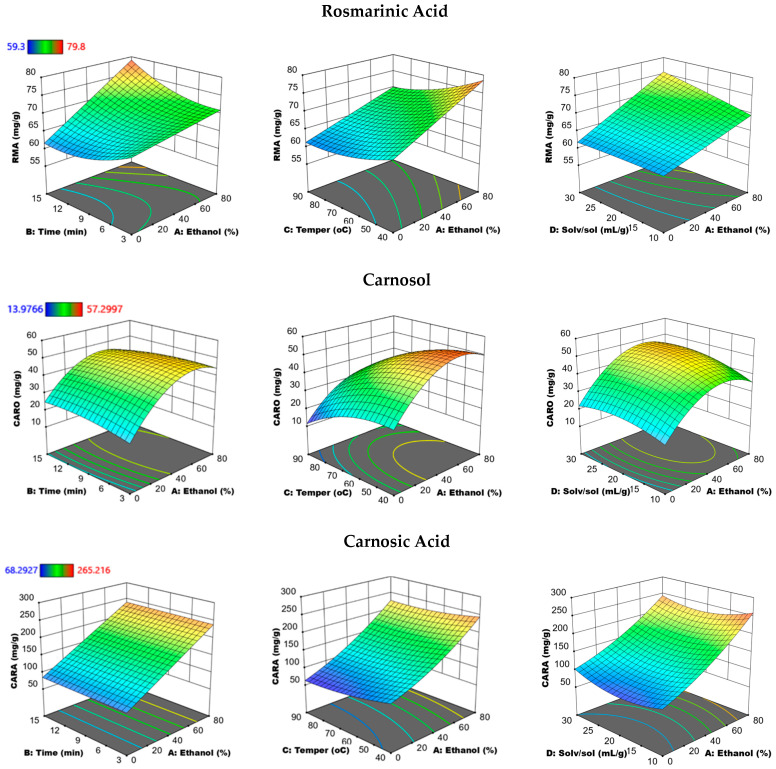
Response surface plots showing the combined effects of the MAE parameters (% ethanol-time, % ethanol-temperature, and % ethanol solvent-to-solid ratio) on rosmarinic acid (RMA), carnosol (CARO), and carnosic acid (CARA) by keeping the two independent variables constant at medium levels.

Three-dimensional response surface graphs were constructed to illustrate the simultaneous effect of two independent variables (all combined with ethanol concentration) on the EY, TPC, and antioxidant activity of phenolic extracts (Figure 2), as well as the main phenolic components such as RMA, CARO, and CARA, keeping the other two independent variables at a certain level obtained by MAE (Figure 3). The relationships between the other parameters (time, temperature, and solvent to solid) on all responses are illustrated in the Supplementary Data (Figures S2 and S3). As demonstrated in Figure 2, the highest EY of phenolics from PRES could be obtained with the lowest ethanol concentration and highest solvent-to-solid ratio. An increase in the extraction temperature conferred either a negative or positive effect on EY. This is evident with the slow linear increase in the EY from 19 to 26% as the temperature rose from 40 to 90 °C, using a solvent-to-solid ratio of 20 mL/g and time of 9 min. These results are in accordance with those of Jacotet-Navaro et al. [39] who found an increase in EY with increasing microwave temperatures; with the highest yield (25.2%) being reached at 150 °C for rosemary leaves.

Figure 2 and Figure 3 show that the TPC, RMA, and ABTS responses have similar trends with respect to solvent concentration. Approximately 80% of the solvent concentration showed the highest efficiency for the extraction of phenolic antioxidants with the highest TPC, RMA, CARA, and ABTS radical scavenging activity, which was in accordance with the study by Jacotet-Navaro et al. [39] using an ethanol concentration of 90% for the MAE of rosemary leaves. Similarly, Alonso-Carrillo et al. [15] found the best ethanol concentration for the MAE of *Satureja macrostema* was 75%. However, an ethanol concentration of approximately 60% showed the best performance in extracting CARO with the greatest DPPH radical scavenging activity. Exceeding this critical value, a decrease occurred in the above responses. 

In addition, Figure 2 and Figure 3 show that a slight decrease in TPC, RMA, CARO, CARA, and ABTS responses occurred with the MAE temperature near 90 °C. However, as the MAE temperature increased from 40 to 60 °C, a significant rise in DPPH response appeared, while any further increase in temperature over 60 °C resulted in a corresponding reduction in DPPH response. As reported in many studies, even if the efficiency of the extraction increased with the MAE temperature, phenolics with antioxidant properties, including RMA, CARO, and CARA, still structurally decompose at high temperatures [42]. 

All responses slightly increased with the extraction time and solvent-to-solid ratio. In the present study, it was noticed that the extraction time was a much less significant factor in the extraction of phenolic antioxidants from PRES compared to solvent concentration and temperature, which was in accordance with the findings of Bener [43] who optimized the MAE of phenolics from *Thymbra Spicata* L. This is an interesting finding, as the MAE system offers the opportunity to extract with high efficiency and in a short time, contributing to energy saving.

### 3.3. Effect of Extraction Factors on Experimental Responses Using UAE

According to the results of Table 4, the recovery of phenolic extracts from PRES using UAE was mainly affected by the linear terms of ethanol concentration and extraction temperature as confirmed by the presence of the significant (*p* ≤ 0.05) respective coefficients in all the response variables tested. The positive coefficient values for ethanol concentration (except for EY) and temperature for almost all the responses reveal that increasing the values of these parameters increases the values of the responses. However, the application of high ethanol concentrations does not always result in an increase in the content of phenolics and antioxidant activity. In almost all the responses, besides the solvent concentration linear term, the quadratic term was also significant, except for the RMA response, suggesting the presence of curvatures in the variation. Ethanol concentrations at about 50% not only decelerate the recovery of TPC, TFC, DPPH, and ABTS but also decrease their values. In the case of CARO, only a slight deceleration was observed at much higher concentrations, whereas CARA exhibited a continuous increase without a sign of deceleration within the range of the concentrations studied. In their study, de Oliveira et al. [44] reported that the RMA, CARO, and CARA recovery in rosemary during conventional extraction (solid-liquid extraction) is favored when the ethanol content is below 70%.

In general, increasing the temperature increases the recovery of phenolics and subsequent antioxidant activity in PRES extracts. An exception is the recovery of CARO and CARA which showed negative temperature coefficient values, suggesting these two phenolic diterpenes are more sensitive to temperature than RMA and that higher recovery levels can be achieved only by applying low temperatures. Contrary to the MAE process, RMA was positively affected by the temperature but was the only response having a significant quadratic effect of temperature. This result indicates that a positive trend was observed until around 45 °C but that a further increase in the temperature decreases its recovery. In general, during MAE, the temperature is a less important parameter compared to UAE. 

The time factor negatively affects EY, but positively affects RMA, CARO, and CARA, as observed by their respective coefficients, suggesting that the longer UAE extraction time resulted in a higher recovery of phenolics. For the EY, the presence of significant quadratic terms suggests a variation in the behavior, with the continuous increase in EY above 6 min of extraction. In addition, a significant interaction effect of time with temperature was observed for the recovery of RMA. The mathematical model suggested that operating UAE at intermediate temperatures (45 °C) could achieve higher RMA recovery values.

Contrary to MAE, EY and CARO in PRES extracts were not affected by the solvent-to-solid ratio factor. In addition, a positive significant linear effect was noticed for the solvent-to-solid ratio in TPC, TFC, ABTS, and DPPH, but not quadratic effects, suggesting that an increase in the ratio increases the recovery of phenolic antioxidants. A significant positive quadratic effect for CARO reveals differences in behavior with increasing the solvent-to-solid ratio. Increasing the ratio to 20 resulted in a slight decrease in the recovery of CARO but a further increase reverted this trend. The presence of the negative interaction terms of the solvent-to-solid ratio with the solvent and the temperature, in the case of TPC, TFC, ABTS, and DPPH, stresses the higher importance of this parameter and its effect on the behavior of the two other parameters (ethanol concentration and temperature). With an ethanol concentration lower than 30% and temperatures lower than 40 °C, it was better to use a high solvent-to-solid ratio, whereas, for higher concentration and temperature levels, a lower ratio was preferred in order to recover high TPC and extracts with high ABTS activity. In the case of TFC and DPPH, the behavior was almost the same, with ethanol concentration and temperature values shifted towards much lower levels. Regarding RMA, the interaction terms of the solvent-to-solid ratio with solvent and temperature show positive values, revealing the opposite behavior with TPC and ABTS. At ethanol concentrations lower than about 20% and temperatures lower than 35 °C, it was better to use a low solvent-to-solid ratio, whereas, for higher concentrations and temperature levels, a higher ratio was needed in order to recover high RMA.

**Figure 4:**
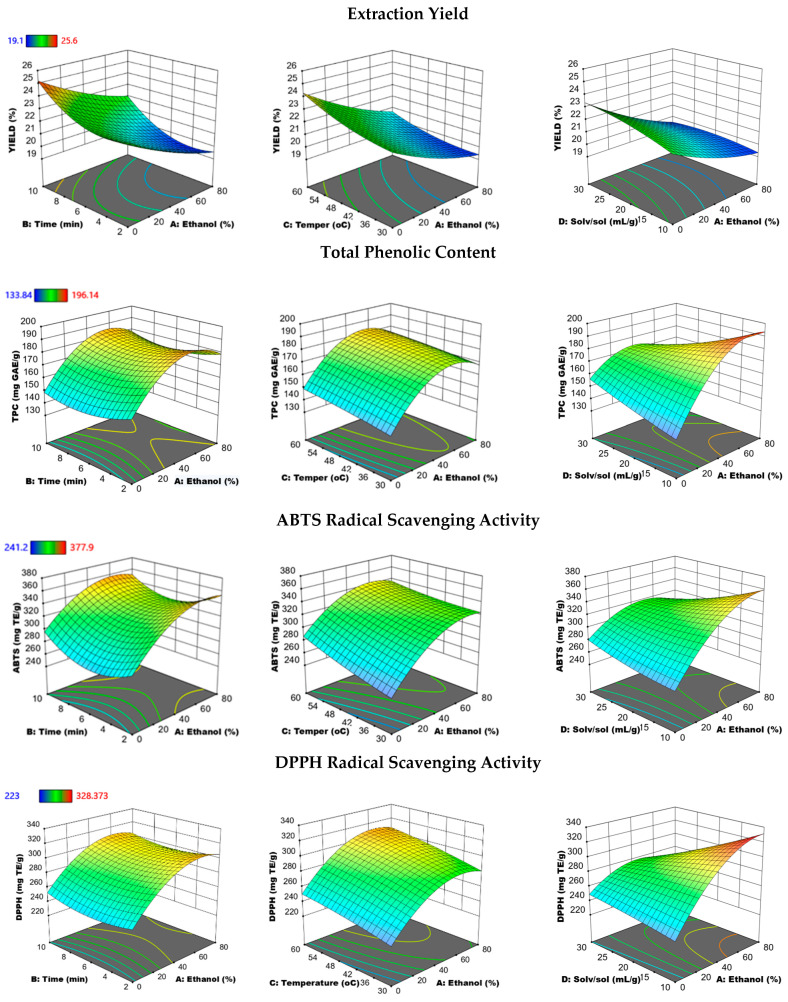
Response surface plots showing the combined effects of the UAE parameters (% ethanol-time, % ethanol-temperature, and % ethanol solvent-to-solid ratio) on the extraction yield, total phenolic content, ABTS, and DPPH radical scavenging activity by keeping the two independent variables constant at medium levels.

**Figure 5:**
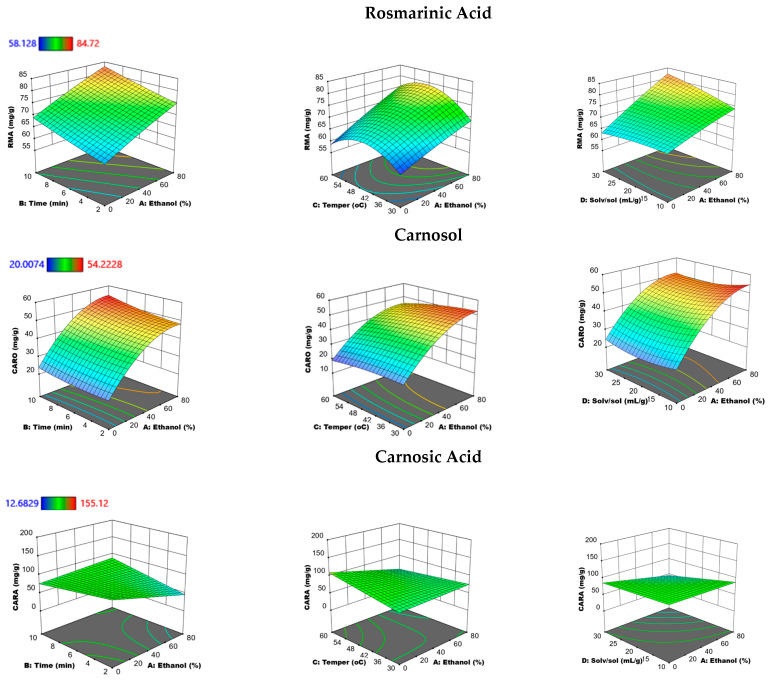
Response surface plots showing the combined effects of the UAE parameters (% ethanol-time, % ethanol-temperature, and % ethanol solvent-to-solid ratio) on rosmarinic acid (RMA), carnosol (CARO), and carnosic acid (CARA) by keeping the two independent variables constant at medium levels.

The 3D response surface plots in Figure 4 and Figure 5 illustrate the relationship between the different factors (all combined with ethanol concentration) and how these factors affect the EY, TPC, RMA, CARO, CARA, ABTS, and DPPH responses by UAE. The relationships between the other parameters (time, temperature, and solvent to solid) on all the above responses are illustrated in the Supplementary Data (Figures S4 and S5). As can be seen in Figure 4, the EY of phenolics from PRES increased with the increase in UAE time and temperature using the lowest ethanol concentration (0%). Thus, the highest EY (26%) was obtained when water was used as a solvent and the time and temperature extraction values were at their highest levels (10 min and 60 °C), using a solvent-to-solid ratio of 20 mL/g. Similarly, when increasing temperature from 30 °C to 65 °C the TPC as well as the ABTS and DPPH radical scavenging activity increases, using 40% ethanol concentration for 6 min UAE. With the increasing UAE temperature, the solubility of polyphenols increases due to the penetration of solvent into the plant matrix and the higher mass transfer rate, as also reported in many studies [43,45].

As we can observe from Figure 5, the RMA content increased as the temperature rose from 30 to 45 °C, however, a further decrease was observed upon increasing the temperature from 45 °C to 60 °C. Moreover, a decrease in the CARO and CARA contents was obtained as the UAE temperature increased. This tendency may be due to the degradation of CARA with the increased temperature. This conclusion is in accordance with the findings of other researchers [38,39], referring to the degradation of CARA even with mild temperatures. However, an enhanced extraction of CARA has been achieved with an increased temperature of 100 to 200 °C using pressurized water extraction [46,47]. An ethanol concentration of approximately 60% showed the highest efficiency for the extraction of phenolic antioxidants with the highest TPC, DPPH, and ABTS radical scavenging activity. However, an approximate 80% ethanol concentration showed the best performance for extracting RMA, CARO, and CARA. 

### 3.4. Optimal Experimental Conditions for MAE and UAE Systems

The MAE and UAE of phenolic antioxidants from PRES were successfully optimized using the regression equations of the RSM presented in Table 4. Optimization was based on the maximum desirability function for the maximum EY, TPC, TFC, RMA, CARO, CARA, ABTS, and DPPH responses. The desirability function consolidates all the responses into one response with a numerical value varying from 0 (one or more product characteristics are unacceptable) to 1 (all product characteristics are on target). In our study, the maximum desirability for the MAE and UAE models were 0.84 and 0.86, respectively. The optimal experimental conditions for ethanol concentration, time, temperature, and solvent-to-solid ratio were 72 and 68%, 15 and 10 min, 40 and 47 °C, and 30 and 10 mL/g for MAE and UAE, respectively. In contrast, Zekovic et al. [3] reported that the optimal MAE parameters that maximized TPC and TFC from sage herbal by-products were 46.2% aqueous ethanol as an extraction solvent, an extraction time of 18.7 min, and a liquid-to-solid ratio of 40 mL/g. This disagreement could be due to the different processing applied to the sage by-products and/or the target phenolic compounds used as responses through the RSM. It was demonstrated that RMA extraction is enhanced using UAE and MAE, as it does not appear to be a thermo-sensitive compound, and this finding is in accordance with Jacotet-Navarro et al. [39] who adopted UAE and MAE processes to extract RMA, CARO, and CARA from rosemary leaves. 

The experimental values for the responses under the optimal conditions for MAE and UAE and the corresponding predicted responses are presented in Table 5. The experimental values for the responses under these conditions did not report significant differences with predicted values, with a CV ranging from 2.24 to 8.63% and from 2.68 to 8.69% for MAE and UAE, respectively. This reveals that both models are well-fitted for the extraction of phenolic antioxidants from PRES under optimal MAE and UAE conditions and that the designed models have good, predicted responses. Statistical analyses of the experimental values between the MAE and UAE responses showed no significant (*p* > 0.05) differences, except for CARA content and DPPH radical scavenging activity. Under the optimal conditions, MAE extracts resulted in 50% more CARA from PRES compared with UAE, but 24% less DPPH radical scavenging activity. The decrease in the DPPH value can be explained by differences in the antioxidant activity of the individual phenolics and/or their interactions in the mixture that can be triggered by changes in the component’s concentration. Thus, we noticed that a higher amount of CARA was extracted by MAE compared to UAE, even at higher extraction temperatures. Similarly, Lesellier et al. [45] found no significant difference in the recovery of RMA from sage when applying MAE (75 °C) or UAE (25 °C) using 75% ethanol. However, they noticed that the ultrasound probe resulted in a higher recovery for CARA, whereas increased temperature favored RMA recovery.

In the paper published by Djarmati et al. [48], the content of CARA obtained by supercritical-CO_2_ was 137.6 mg/g of the extract, and the CARO content was 69.7 mg/g of the extract, similar to our results (49.6 mg/g CARO and 108.3 mg/g CARA). It has been reported that CARA is oxidized to CARO during the degradation pathway of CARA in methanol or ethanol after the extraction from plant material, whereas the RMA seemed to be stable in the ethanol solution [38]. Similar to our results, Jacotet-Navarro et al. [39] concluded that the decrease in CARA in extracts does not result in an increase in CARO, but rather an increase in other minor degradation derivatives of CARA such as epirosmanol [49] (and probably rosmanol derivatives [39]). Altogether, our results indicate that higher pressure and intensification through the US probe favor a higher ratio of CARO compared to CARA.

## 4. Conclusions

In the present work, the optimization and comparison of two ‘green’ extraction processes (MAE and UAE) were studied for the recovery of phenolic compounds from the *Salvia fruticosa* L. solid waste that remains after the essential oil distillation. The obtained phenolic extracts presented high TPC and TFC and were rich in phenolics such as RMA, CARO, and CARA. It was observed that CARA extraction is enhanced using microwave processes. In addition, a higher solvent quantity and higher ethanol concentration along with a lower extraction temperature are required in the case of MAE compared to UAE for optimum extracts in terms of phenolic content and antioxidant activity. According to the results presented in this study, it could be concluded that PRES can be considered a sustainable raw material for the recovery of phenolic extracts through UAE or MAE. In addition, these findings revealed that UAE and MAE have both advantages and disadvantages but represent good alternative processes in terms of lower energy requirements and shorter extraction times. For the industrial application, this analytical approach could be a basis for further pilot-scale trials of UAE and MAE as green extraction technologies for the recovery of high-added value phenolic extracts from the post-distillation residues of the essential oil industry. Therefore, the derived phenolic extracts from PRES could serve as a sustainable and functional ingredient for further application in the food and pharmaceutical industries, instead of being discarded as a by-product. 

## Figures and Tables

**Table 1:** Coded and actual levels of the independent variables used for ultrasound-assisted extraction (UAE) and microwave-assisted extraction (MAE).

**UAE**	**Coded Values**		
	**−1**	**0**	**+1**
Ethanol concentration (%)	0	40	80
Extraction time (min)	2	6	10
Extraction temperature (°C)	30	45	60
Solvent-to-solid ratio (mL/g)	10	20	30
**MAE**	**Coded Values**		
	**−1**	**0**	**+1**
Ethanol concentration (%)	0	40	80
Extraction time (min)	3	9	15
Extraction temperature (°C)	40	65	90
Solvent-to-solid ratio (mL/g)	10	20	30

**Table 2:** Box–Behnken experimental design with the observed responses for the microwave-assisted extraction (MAE) and ultrasound-assisted extraction (UAE) from sage solid residue.

Run	Independent Variables				Response Variables							
	X_1_	X_2_	X_3_	X_4_	EY	TPC	TFC	RMA	CARO	CARA	ABTS	DPPH
**Microwave-Assisted Extraction**												
1	0	3	65	20	20.8	147.4	240.6	69.6	15.8	95.9	266.4	222.4
2	80	3	65	20	18.6	182.3	261.9	74.2	40.1	248.4	337.1	258.5
3	0	15	65	20	27.0	153.4	230.1	62.0	19.9	82.7	268.9	213.0
4	80	15	65	20	21.4	189.4	280.9	79.8	35.4	265.2	352.2	265.6
5	40	9	40	10	16.1	183.5	264.0	73.7	34.3	204.3	342.4	251.5
6	40	9	90	10	18.4	175.8	245.7	65.7	23.8	152.9	288.8	269.1
7	40	9	40	30	22.6	199.6	271.2	75.7	45.4	178.2	295.9	261.4
8	40	9	90	30	24.4	178.1	256.7	67.0	28.0	166.2	285.9	245.4
9	0	9	65	10	21.7	150.6	243.0	59.3	14.0	91.1	249.6	213.6
10	80	9	65	10	15.4	181.7	280.8	66.4	36.4	240.6	325.9	275.7
11	0	9	65	30	22.1	153.4	219.8	61.9	20.4	115.1	239.2	201.8
12	80	9	65	30	18.2	196.1	254.9	75.2	49.7	238.8	303.9	250.2
13	40	3	40	20	22.0	187.7	241.9	73.4	48.9	171.6	305.2	261.8
14	40	15	40	20	22.5	194.4	268.3	75.4	57.3	187.2	326.6	262.5
15	40	3	90	20	23.6	178.6	248.4	62.4	27.6	142.7	281.7	241.0
16	40	15	90	20	23.1	178.2	250.5	67.6	22.1	113.3	300.3	258.2
17	0	9	40	20	24.4	137.9	229.0	63.2	34.3	75.5	240.0	210.8
18	80	9	40	20	19.0	195.8	268.1	76.0	49.6	226.2	311.8	261.8
19	0	9	90	20	24.2	165.6	218.4	63.4	21.9	68.3	261.8	207.5
20	80	9	90	20	20.8	203.0	246.2	72.2	30.8	237.0	305.9	248.7
21	40	3	65	10	19.3	177.9	257.8	67.7	48.7	166.2	292.2	265.0
22	40	15	65	10	18.5	159.8	242.4	69.2	43.8	174.0	324.7	289.6
23	40	3	65	30	23.0	188.0	233.7	69.2	45.1	156.5	273.9	286.0
24	40	15	65	30	26.4	185.2	242.6	70.8	45.9	163.2	328.9	274.2
25	40	9	65	20	20.5	182.3	242.4	66.0	45.6	136.7	305.6	283.7
26	40	9	65	20	18.5	176.4	248.5	66.3	49.1	145.7	317.1	275.0
27	40	9	65	20	19.0	179.4	242.9	69.8	45.4	121.1	304.0	283.8
**Ultrasound-Assisted Extraction**												
1	0	2	45	20	23.6	154.2	213.0	59.3	22.3	34.9	285.7	247.8
2	80	2	45	20	19.1	184.2	231.6	77.2	49.3	98.6	358.5	307.7
3	0	10	45	20	25.6	149.7	213.1	65.1	23.0	63.0	291.7	254.7
4	80	10	45	20	21.2	185.2	248.2	83.5	54.2	155.1	346.5	308.2
5	40	6	30	10	20.5	174.1	234.7	65.6	47.8	91.2	303.5	289.9
6	40	6	60	10	21.2	196.1	265.5	63.7	45.7	65.0	377.9	320.9
7	40	6	30	30	20.2	184.1	230.7	59.2	45.0	90.3	320.4	279.9
8	40	6	60	30	22.1	171.1	220.9	68.5	41.4	63.3	314.3	278.3
9	0	6	45	10	21.1	139.3	206.3	64.4	21.8	33.5	259.0	239.1
10	80	6	45	10	19.7	190.4	247.4	72.8	51.0	125.4	347.0	328.4
11	0	6	45	30	22.7	159.7	210.0	65.9	26.2	45.9	293.3	254.0
12	80	6	45	30	20.0	150.3	218.8	84.7	51.6	130.5	300.9	254.2
13	40	2	30	20	20.7	182.9	257.2	58.1	45.0	75.0	346.1	287.0
14	40	10	30	20	21.4	178.1	246.3	71.0	48.4	80.0	331.1	285.8
15	40	2	60	20	22.5	189.3	266.2	67.8	37.1	60.0	354.6	321.0
16	40	10	60	20	24.4	184.5	255.6	69.9	38.8	73.5	368.6	310.6
17	0	6	30	20	22.6	133.8	187.7	60.6	22.2	34.4	241.2	223.0
18	80	6	30	20	19.8	162.6	212.7	67.0	53.4	138.3	334.4	280.3
19	0	6	60	20	24.3	148.1	207.9	60.3	20.0	12.7	276.1	245.2
20	80	6	60	20	20.7	175.0	220.9	73.0	46.8	123.3	341.5	308.0
21	40	2	45	10	19.8	185.9	253.0	65.9	47.5	72.3	343.7	310.7
22	40	10	45	10	23.0	190.9	266.0	75.0	52.6	84.0	375.1	315.0
23	40	2	45	30	20.4	180.5	242.7	68.0	42.0	60.3	335.1	280.7
24	40	10	45	30	24.8	170.1	247.4	78.9	50.0	87.9	349.2	285.1
25	40	6	45	20	21.0	179.2	237.8	72.0	45.0	69.0	318.8	290.9
26	40	6	45	20	21.2	180.6	238.6	70.4	42.5	75.6	328.7	291.7
27	40	6	45	20	20.6	173.9	230.7	74.7	43.3	76.2	317.4	298.4

X_1_: ethanol concentration (%); X_2_: time extraction (min); X_3_: temperature extraction (°C); X_4_: solid/solvent ratio (g/L); EY: extraction yield (%); TPC: total phenolic content (mg GAE/g); TFC: total flavonoid content (mg CATE/g); RMA: rosmarinic acid (mg/g); CARO: carnasol (mg/g); CARA: carnosic acid (mg/g); ABTS: ABTS radical scavenging activity (mg TE/g); and DPPH: DPPH radical scavenging activity (mg TE/g).

**Table 3:** Identification of the main peaks in sage solid residue.

Retention Time (min)	UV *λ*max (nm)	[M-H]^−^ (m/z)	Other Fragments	Compound
12.2	329, 285sh	359	405, 161	Rosmarinic acid
33.7	280	329	285	Carnosol
37.0	280	331	287	Carnosic acid

**Table 4:** Polynomial regression equations for the eight response variables (EY, TPC, TFC, RMA, CARO, CARA, ABTS, and DPPH) of the post-distillation solid residue of sage extract using MAE and UAE.

Response	Polynomial Regression Equations in Uncoded Units *	R^2^	R^2^ (adj)	R^2^ (pred)	Lack-of-Fit	
					F	p
	**Microwave-Assisted Extraction**					
EY	Y = 28.91 − 0.0555X_1_ − 0.896X_2_ − 0.283X_3_ * + 0.02271X_4_ + 0.0587X_2_^2^ + 0.002382X_3_^2^	0.79	0.73	0.61	2.23	0.36
TPC	Y = 139.52 + 1.014X_1_ + 0.0593X_4_ − 0.00643X_1_^2^	0.80	0.77	0.72	8.11	0.12
TFC	Y = 248.22 + 0.4414X_1_ − 0.255X_3_	0.61	0.58	0.50	10.75	0.09
RMA	Y = 76.02 + 0.0104X_1_ − 0.435X_2_ * − 0.1303X_3_ + 0.01375X_1_X_2_	0.73	0.68	0.55	2.08	0.38
CARO	Y = 46.05 + 0.744X_1_ − 0.3849X_3_ − 0.00628X_1_^2^	0.73	0.70	0.63	11.64	0.08
CARA	Y= 178.5 + 1.932X_1_ − 0.541X_3_ − 0.652X_4_ * + 0.001608X_4_^2^	0.92	0.90	0.88	2.10	0.37
ABTS	Y = 236.18 + 1.672X_1_ + 2.015X_2_ − 0.01020X_1_^2^	0.74	0.70	0.63	5.81	0.16
DPPH	Y = 118.5 + 2.336X_1_ + 3.156X_3_ * − 0.02161X_1_^2^ − 0.02530X_3_^2^	0.88	0.86	0.82	4.02	0.22
**Ultrasound-Assisted Extraction**						
EY	Y = 20.82 − 0.04042X_1_ − 0.473X_2_ + 0.0556X_3_ + 0.0642X_2_^2^	0.82	0.79	0.72	7.42	0.13
TPC	Y = 77.1 + 2.051X_1_ − 5.06X_2_ * + 1.436X_3_ + 0.3625X_4_ − 0.01195X_1_^2^ + 0.39X_2_^2^ − 0.003776X_1_X_4_ − 0.00583X_3_X_4_	0.95	0.92	0.86	1.87	0.40
TFC	Y = 157.2 + 1.948X_1_ − 11.21X_2_ * + 1.729X_3_ + 0.3X_4_ − 0.01564X_1_^2^ + 0.956X_2_^2 −^ 0.002012X_1_X_4_ − 0.00676X_3_X_4_	0.91	0.88	0.77	3.11	0.27
RMA	Y = 2.60 + 0.0413X_1_ +3.009X_2_ + 2.593X_3_ − 0.0954X_4_ * − 0.02863X_3_^2^ + 0.000656X_1_X_4_ − 0.0451X_2_X_3_ + 0.001867X_3_X_4_	0.92	0.88	0.79	1.35	0.51
CARO	Y = 38.92 + 0.7292X_1_ + 0.498X_2_ − 0.1774X_3_ − 0.1138X_4_ * − 0.00466X_1_^2^ + 0.000263X_4_^2^	0.96	0.95	0.93	3.69	0.24
CARA	Y= 47.47 + 0.735X_1_ + 2.967X_2_ − 0.619X_3_ + 0.00505X_1_^2^	0.94	0.93	0.89	5.77	0.16
ABTS	Y = 129.8 + 3.2X_1_ − 16.65X_2_ * + 3.552X_3_ + 0.727X_4_ − 0.0175X_1_^2^ + 1.454X_2_^2 −^ 0.00503X_1_X_4_ − 0.01342X_3_X_4_	0.94	0.91	0.84	3.02	0.28
DPPH	Y = 161.7 + 2.969X_1_ − 6.90X_2_ * + 1.859X_3_ + 0.3252X_4_ − 0.01477X_1_^2^ + 0.583X_2_^2^ − 0.005568X_1_X_4_ − 0.00546X_3_X_4_	0.97	0.96	0.93	1.79	0.42

X_1_, ethanol concentration; X_2_, time extraction; X_3_, temperature extraction; X_4_, solvent-to-solid ratio; EY, extraction yield (%); TPC. total phenolic content (mg GAE/g); TFC. total flavonoid content (mg CATE/g); RMA. rosmarinic acid (mg/g); CARO, carnasol (mg/g); CARA, carnosic acid (mg/g); ABTS, ABTS radical scavenging activity (mg TE/g); and DPPH, DPPH radical scavenging activity (mg TE/g). * Only significant equation terms were reported except for main terms if coefficients that explain quadratic or interaction effects were significant (*p* ≤ 0.05)

**Table 5:** Optimal conditions obtained by the response surface methodology for microwave-assisted extraction (MAE) and ultrasound-assisted extraction (UAE) as well as the experimental and predicted values of the investigated responses.

Extraction Method	Optimized Conditions	Response	Predicted Values	Experimental Values	% Difference (CV)
UAE	Ethanol: 67.9% Time: 10 min Temperature: 47 °C Solvent/solid: 10 mL/g Desirability: 0.84	Yield (%)	22.38 ± 0.36	23.73 ± 0.23 A	5.98
		TPC (mg GAE/g)	199.57 ± 3.03	191.77 ± 1.40 A	3.05
		TFC (mg CATE/g)	266.07 ± 4.66	272.21 ± 2.43 A	2.68
		RMA (mg/g)	76.81 ± 1.53	79.57 ± 4.10 A	4.64
		CARO (mg/g)	54.59 ± 1.24	49.59 ± 3.10 A	7.19
		CARA (mg/g)	121.22 ± 3.72	108.26 ± 7.95 B	8.69
		ABTS (mg TE/g)	386.73 ± 6.55	367.84 ± 7.63 A	3.81
		DPPH (mg TE/g)	339.57 ± 3.43	344.74 ± 11.92 A	2.70
MAE	Ethanol: 71.6% Time: 15 min Temperature: 40 °C Solvent/solid: 30 mL/g Desirability: 0.86	Yield (%)	23.98 ± 1.02	24.50 ± 0.61 A	5.87
		TPC (mg GAE/g)	196.97 ± 3.42	190.30 ± 6.62 A	3.99
		TFC (mg CATE/g)	269.61 ± 4.42	273.57 ± 1.43 A	2.24
		RMA (mg/g)	79.80 ± 1.91	78.76 ± 2.71 A	3.89
		CARO (mg/g)	51.69 ± 2.84	48.44 ± 2.01 A	8.63
		CARA (mg/g)	244.25 ± 9.68	222.96 ± 10.31 A	7.89
		ABTS (mg TE/g)	333.84 ± 6.99	358.90 ± 7.70 A	4.97
		DPPH (mg TE/g)	260.76 ± 4.71	277.77 ± 6.05 B	4.39

Different upper case letters in the same column for the same response indicate significant differences (*p* ≤ 0.05) among the means, according to Tukey’s test.

## Data Availability

Data is contained within the article or Supplementary Materials.

## Supplementary Materials

The following supporting information can be downloaded at: https://www.mdpi.com/article/10.3390/antiox12030549/s1, Table S1: Analysis of variance for response surface models for the microwave-assisted extraction and ultrasound-assisted extraction, showing linear, quadratic and interaction relations of each variable and coefficients; Figure S1: Main effects of the MAE and UAE parameters (% ethanol, time and temperature extraction as well as solvent/solid ratio) for extraction yield (EY), total phenolic content (TPC), total flavonoid content (TFC), rosmarinic acid (RMA), carnosol (CARO), carnosic acid (CARA), ABTS and DPPH radical scavenging activity; Figure S2. Response surface plots showing combined effects of the MAE parameters (time-temperature, time-solvent/solid, temperature-solvent/solid) for extraction yield (EY), total phenolic content (TPC), ABTS and DPPH radical scavenging activity; Figure S3: Response surface plots showing combined effects of the MAE parameters time-temperature, time-solvent/solid, temperature-solvent/solid) for rosmarinic acid (RMA), carnosol (CARO), and carnosic acid (CARA); Figure S4: Response surface plots showing combined effects of the UAE parameters (time-temperature, time-solvent/solid, temperature-solvent/solid) for extraction yield (EY), total phenolic content (TPC), ABTS and DPPH radical scavenging activity; Figure S5: Response surface plots showing combined effects of the UAE parameters time-temperature, time-solvent/solid, temperature-solvent/solid) for rosmarinic acid (RMA), carnosol (CARO) and carnosic acid (CARA).
