# Supplementary material for: Modeling and Optimization of Phenolic Compounds from Sage (Salvia fruticosa L.) Post-Distillation Residues: Ultrasound- versus Microwave-Assisted Extraction
Source: Antioxidants (Basel). 2023 Feb 21;12(3):549. doi: 10.3390/antiox12030549 (PMC10045014; doi:10.3390/antiox12030549)
Supplement: Supplementary file 1 [file antioxidants-12-00549-s001.zip › antioxidants-2222054-supplementary.pdf]

SUPPLEMENTARY DATA

Table S1. Analysis of variance for response surface models for the microwave-assisted extraction and ultrasound-assisted extraction, showing linear, quadratic and interaction relations of each variable and coefficients.

Coefficients	EY	TPC	TFC	RMA	CARO	CARA	ABTS	DPPH
**Microwave-assisted extraction**								
**Intercept, β_0**	19.33	179.36	244.58	67.37	46.67	134.53	308.90	208.83
**Linear**								
β_1	-2.22***	20.01***	17.66***	5.37***	9.64***	77.30***	34.24***	24.28***
β_2	0.96	-0.14	2.54	0.69	-0.16	0.35	12.09*	2.37
β_3	0.65	-1.63	-6.39	-3.26**	-9.62***	-13.53*	-8.13	-3.33
β_4	2.27***	5.93*	-4.57	1.48	2.79	-0.92	-7.99	-3.79
**Quadratic**								
β_1^2	0.63	-9.42*	-0.43	0.01	-12.28**	15.80	-17.83*	-36.52***
β_2^2	2.20**	-1.20	2.72	2.27	-1.98	13.55	6.60	-3.00
β_3^2	1.57*	5.79	3.88	1.57	-5.30	8.11	-7.38	-17.76***
β_4^2	-0.30	-0.28	4.41	-0.17	-3.88	23.56**	-6.75	-4.78
**Interaction**								
β_{12}	-0.86	0.28	7.39	3.30*	-2.18	7.50	3.15	4.12
β_{13}	0.51	-5.13	-2.82	-1.00	-1.58	4.51	-6.93	-2.45
β_{14}	0.61	2.90	-0.68	1.55	1.72	-6.45	-2.90	-3.43
β_{23}	-0.25	-1.77	-6.08	0.80	-3.48	-11.26	-0.70	4.13
β_{24}	1.03	3.82	6.06	0.03	1.42	-0.30	5.62	-9.10
β_{34}	-0.12	-3.46	0.95	-0.18	-1.72	9.83	10.90	-8.40
Mean	21.15	177.09	249.28	69.00	36.26	161.65	297.63	253.25
C.V. (%)	7.69	5.09	4.70	4.36	20.61	10.88	5.18	3.44
R^2	0.85	0.87	0.76	0.85	0.83	0.96	0.88	0.95
R^2 (adj)	0.69	0.72	0.49	0.67	0.63	0.90	0.74	0.89
F-value (model)	5.05*	5.69**	2.75*	4.80**	4.20**	18.18***	6.34***	15.60***
F-value (Lack-of-fit)	2.73	10.88	14.29	2.23	15.52	2.19	5.38	3.37
**Ultrasound-assisted extraction**								
**Intercept, β_0**	20.93	177.90	235.71	72.35	43.59	78.95	321.63	293.66
**Linear**								
β_1	-1.62***	13.57***	11.80***	6.90***	14.24***	-11.61	31.82***	26.92***
β_2	1.19***	-1.54	1.07	3.92***	1.99*	3.44	3.21	0.38
β_3	0.83**	4.05*	5.64*	1.79*	-2.66**	4.97	13.03***	11.51***
β_4	0.41	-5.09**	-8.53**	1.48	-0.85	-12.71	-7.75*	-14.31***
**Quadratic**								
β_1^2	0.36	-19.05***	-24.14***	-0.52	-7.53***	13.37	-26.15***	-24.48***
β_2^2	1.12**	6.31*	16.18***	0.23	1.03	-12.58	25.11***	8.49**
β_3^2	0.36	-0.64	-0.57	-6.63***	-1.30	-10.75	3.09	-2.07
β_4^2	-0.25	0.93	4.08	-0.67	2.57*	8.90	4.30	-1.29
**Interaction**								
β_{12}	0.03	1.34	4.12	0.13	1.07	17.69	-4.50	-1.58
β_{13}	-0.20	-0.47	-3.02	1.59	-1.08	-13.81	-6.95	1.38
β_{14}	-0.33	-15.10***	-8.05	2.62	-0.94	-8.48	-20.10**	-22.27***
β_{23}	0.30	0.02	0.06	-2.71	-0.41	9.06	7.25	-2.30
β_{24}	0.30	-3.87	-2.07	0.45	0.70	-40.37*	-4.33	0.04
β_{34}	0.30	-8.74**	-10.15*	2.80	-0.37	13.13	-20.12**	-8.19*
Mean	21.64	172.37	233.73	68.98	41.26	78.48	324.46	285.06
C.V. (%)	3.81	3.04	3.34	4.04	6.04	8.07	3.13	2.17
R^2	0.89	0.95	0.94	0.93	0.98	0.95	0.96	0.98
R^2 (adj)	0.77	0.90	0.86	0.84	0.95	0.90	0.91	0.95
F-value (model)	7.34**	18.38***	12.39***	11.04***	35.50***	5.77**	20.50***	37.23***
F-value (Lack-of-fit)	8.52	2.39	3.72	1.82	4.34	8.70	3.05	2.50

Effects are statistically significant at p * ≤ 0.05 or p ** ≤ 0.01 or p *** ≤ 0.001. (Abbreviations: β_1, ethanol concentration; β_2, time extraction; β_3, temperature extraction; β_4, solvent-to-solid ratio; EY, extraction yield; TPC. total phenolic content (mg GAE/g); TFC. total flavonoid content (mg CATE/g); RMA. rosmarinic acid (mg/g); CARO. carnasol (mg/g); CARA. carnosic acid (mg/g); ABTS. ABTS radical scavenging activity (mg TE/g); DPPH. DPPH radical scavenging activity (mg TE/g)

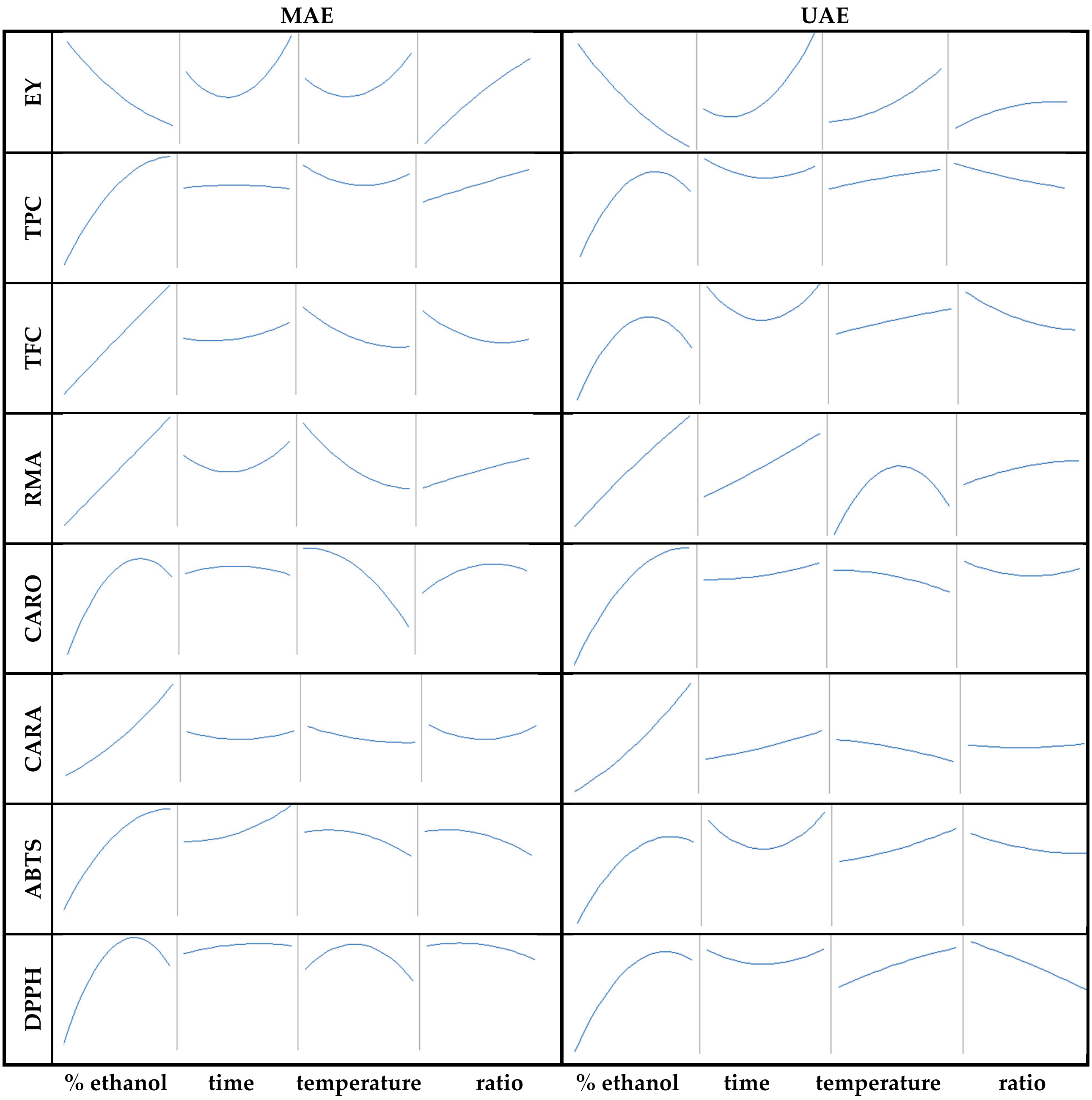

Figure S1. Main effects of the MAE and UAE parameters (% ethanol, time and temperature extraction as well as solvent/solid ratio) for extraction yield (EY), total phenolic content (TPC), total flavonoid content (TFC), rosmarinic acid (RMA), carnosol (CARO), carnosic acid (CARA), ABTS and DPPH radical scavenging activity.

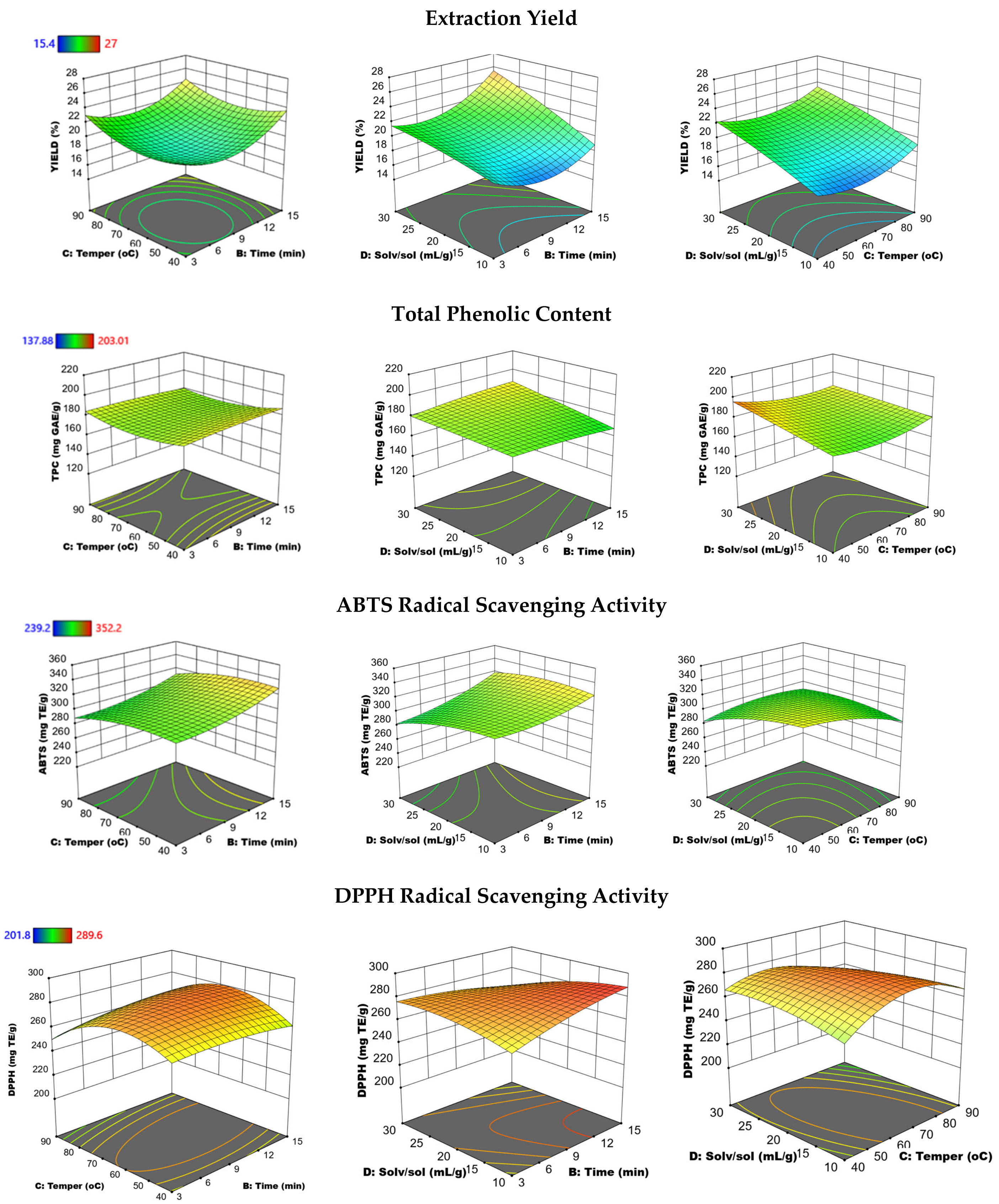

Figure S2. Response surface plots showing combined effects of the MAE parameters (time-temperature, time-solvent/solid, temperature-solvent/solid) for extraction yield (EY), total phenolic content (TPC), ABTS and DPPH radical scavenging activity, by keeping the two independent variables constant at medium levels.

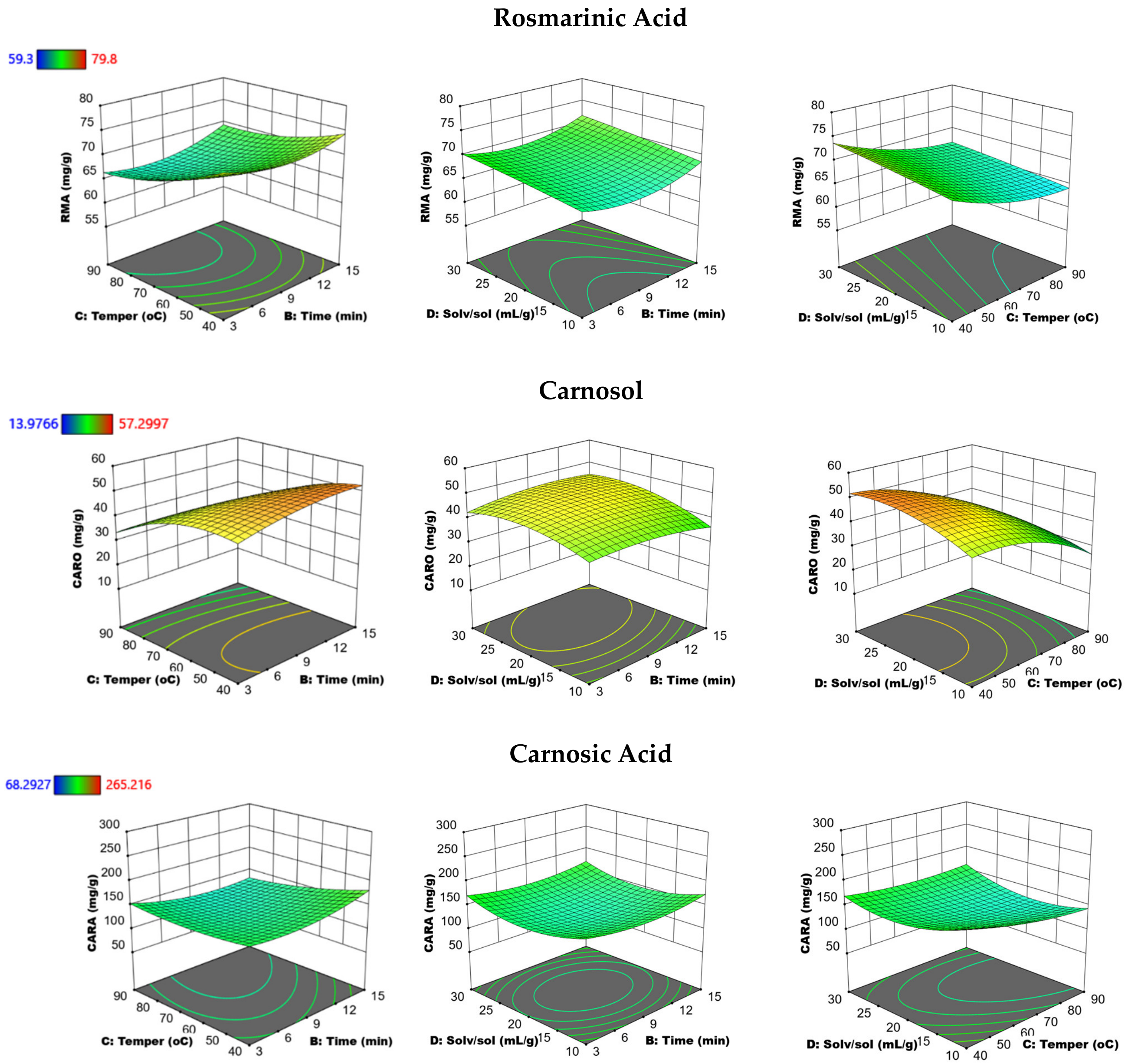

Figure S3. Response surface plots showing combined effects of the MAE parameters time-temperature, time-solvent/solid, temperature-solvent/solid) for rosmarinic acid (RMA), carnosol (CARO) and carnosic acid (CARA), by keeping the two independent variables constant at medium levels.

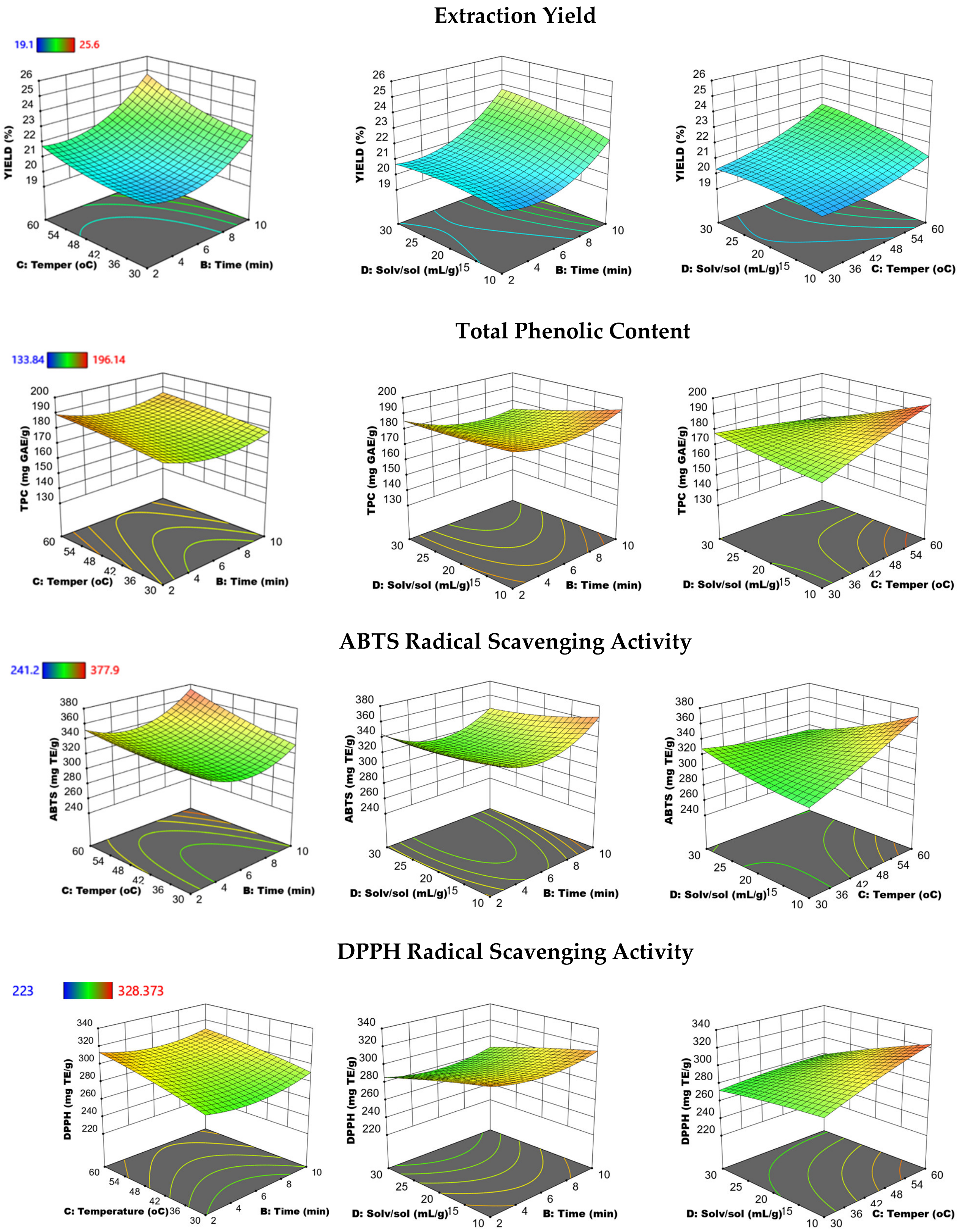

Figure S4. Response surface plots showing combined effects of the UAE parameters (time-temperature, time-solvent/solid, temperature-solvent/solid) for extraction yield (EY), total phenolic content (TPC), ABTS and DPPH radical scavenging activity, by keeping the two independent variables constant at medium levels.

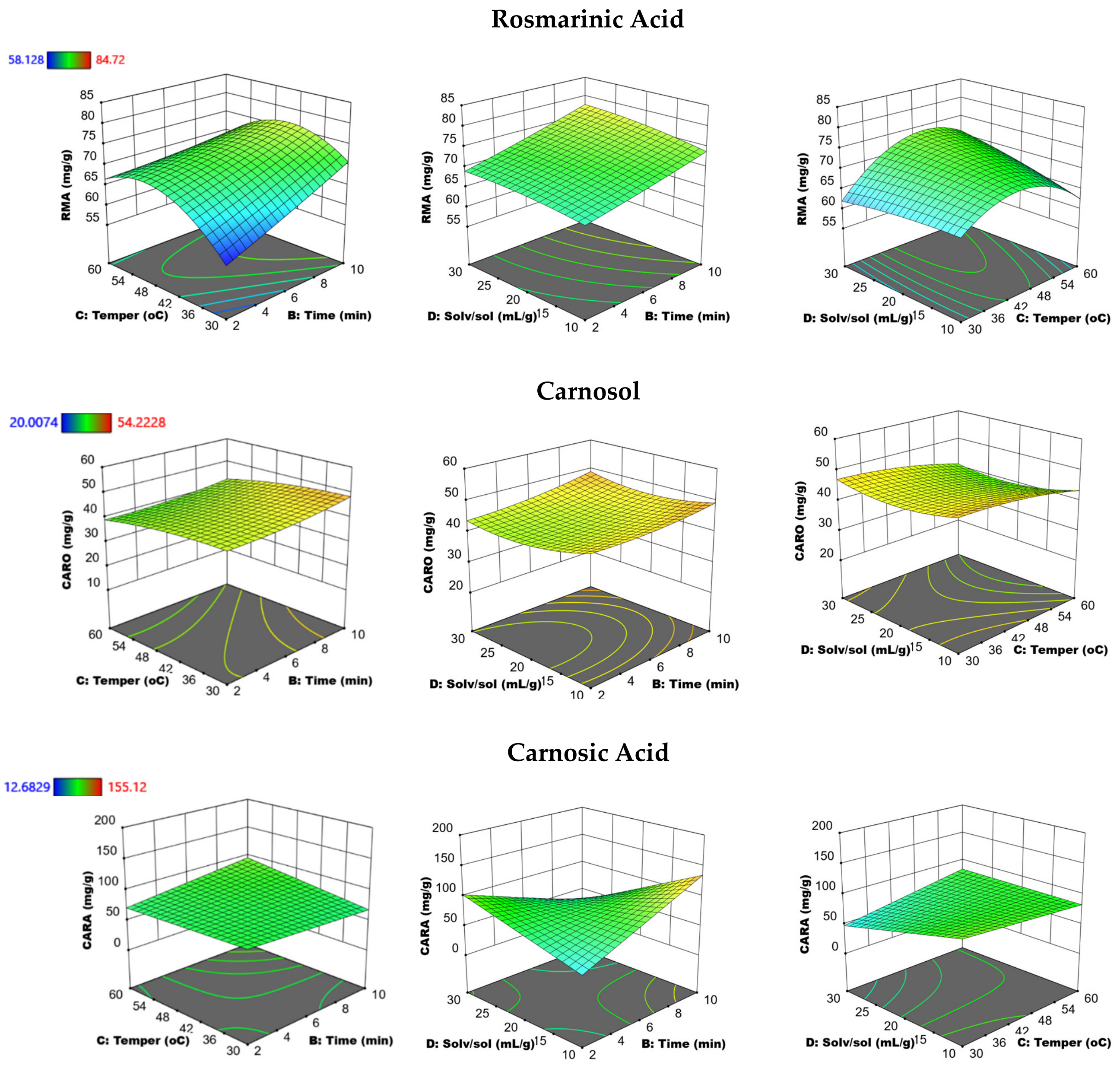

Figure S5. Response surface plots showing combined effects of the UAE parameters time-temperature, time-solvent/solid, temperature-solvent/solid) for rosmarinic acid (RMA), carnosol (CARO) and carnosic acid (CARA), by keeping the two independent variables constant at medium levels.
